# The Role of Gender in the Preconscious Processing of Facial Trustworthiness and Dominance

**DOI:** 10.3389/fpsyg.2019.02565

**Published:** 2019-11-15

**Authors:** Haiyang Wang, Shuo Tong, Junchen Shang, Wenfeng Chen

**Affiliations:** ^1^College of Psychology, Liaoning Normal University, Dalian, China; ^2^Department of Psychology, Renmin University of China, Beijing, China

**Keywords:** preconscious processing, gender, dominance, trust, continuous flash suppression

## Abstract

The present study adopted the breaking continuous flash suppression paradigm (b-CFS) to investigate how Chinese participants process trustworthiness (Experiment 1) and dominance (Experiment 2) at the preconscious level. In addition, we tested whether the gender of a face and the gender of a participant can influence the preconscious processing of facial trustworthiness and dominance. Experiment 1 showed that the least and most trustworthy faces both took significantly less time to break into awareness than neutral faces. In Experiment 2, for female faces, neutral faces took significantly less time to break into awareness than the least and most dominant faces. In both experiments, female faces broke through suppression faster than male faces. In summary, for Chinese participants, the preconscious processing of trustworthiness was not different between male and female faces. However, the preconscious processing of dominance was different between male and female faces.

## Introduction

“Is this person good or bad?” Whenever we meet a stranger, this question is the common issue that must be considered. Our social evaluation of others plays an important role in human evolution. To successfully survive in society, when encountering a stranger, we must quickly judge whether this person is nice or malicious and then whether this person is able to enact his or her intentions (Fiske et al., 2007). Faces are a common part of everyday life; they can convey a range of information such as gender, race, age, and mood. Moreover, many traits can be inferred from faces, such as trustworthiness and dominance. Therefore, our evaluation of others largely depends on their faces (Todorov et al., 2008b). Oosterhof and Todorov (2008) conducted a principal component analysis (PCA) based on observers’ evaluation of multiple traits of neutral faces, identifying two orthogonal dimensions that represent the social traits of faces: trustworthiness and dominance. Trustworthiness is based on facial appearance such as face width, brow ridge, cheek protuberance, and chin shape (Todorov et al., 2008a; Stirrat and Perrett, 2010; Dzhelyova et al., 2012). Trustworthiness is related to one’s intentions and can be used to determine whether to approach or avoid someone. Dominance signals physical strength and ability, representing whether a stranger is dominant or submissive and whether the person is capable of causing harm or the person would be a mighty companion (Todorov et al., 2008b; Hehman et al., 2015).

Dominance and trustworthiness are two main social dimensions of face evaluation, and they are fundamental to social interaction. For instance, an election outcome can be predicted by dominance (Laustsen and Petersen, 2016). Moreover, an officer’s facial dominance was positively correlated with the number of promotions he had received in his career (Mueller and Mazur, 1996). People also tend to approach trustworthy faces but avoid untrustworthy faces (Todorov et al., 2008a). In economic decision-making tasks, people were more willing to give money to people with trustworthy faces (Wout and Sanfey, 2008; Stirrat and Perrett, 2010; Rezlescu et al., 2012). In legal trials, especially in regard to felony convictions, defendants with untrustworthy faces needed less evidence to be convicted than those with trustworthy faces (Porter et al., 2010).

The premise underlying the influence of facial trustworthiness and dominance on advanced social behaviors such as campaigning and decision-making is that faces are perceived by humans. In most studies, faces were presented consciously. However, since we encounter a large number of strangers every day, we cannot always consciously judge their faces. Previous studies have shown that people can make quick judgments about facial traits without conscious perception. Even with a 100-ms exposure to a face, one can judge attractiveness, cuteness, trustworthiness, competence and aggressiveness from facial appearance (Willis and Todorov, 2006). Furthermore, Todorov et al. (2009) found that participants were able to perceive trustworthiness even if the face was presented for only 33 ms. Moreover, 7-month-old babies were able to judge the trustworthiness of faces presented for only 50 ms (Jessen and Grossmann, 2017). In addition, people needed only 40 ms to make judgments of dominance from faces (Rule et al., 2012). In recent years, a number of studies have applied the breaking continuous flash suppression paradigm (b-CFS; e.g., Jiang et al., 2007) to investigate the preconscious processing of faces, demonstrating that facial trustworthiness and dominance could still be processed preconsciously albeit inconsistently (Stewart et al., 2012; Getov et al., 2015; Abir et al., 2018; Stein et al., 2018). In b-CFS, noise patterns were presented to one of the participants’ eyes while faces were presented to the other eye. Participants are asked to press a key on a standard keyboard as soon as any part of the face is detected. The time from the onset of stimulus to the moment it is detected is recorded as the suppression time, which reflects the processing speed of participants under the preconscious condition. Stewart et al. (2012) used computer-generated faces to explore the suppression time by manipulating different levels of dominance and trustworthiness. They found that compared with neutral faces, the most dominant faces and the least trustworthy faces took significantly more time to break through suppression. The findings regarding dominance were replicated by Getov et al. (2015), who used the same stimuli and procedure. However, only a marginally significant difference in suppression times between the least trustworthy faces and neutral faces was found. Moreover, Stein et al. (2018) replicated the findings of Stewart et al. (2012) but suggested that the effect of facial dominance on suppression times was due to low-level physical stimulus characteristics. In addition, Abir et al. (2018) found that more dominant faces and untrustworthy faces took shorter suppression times.

The above studies (Stewart et al., 2012; Getov et al., 2015; Abir et al., 2018; Stein et al., 2018) all used computer-generated faces to investigate the preconscious processing of facial dominance and trustworthiness. Although they could accurately manipulate the experimental combinations with different levels of dominance and trustworthiness, their computer-generated faces were bald males, and they seemed unnatural, reducing the ecological validity of these studies. Moreover, the artificial faces were generated by FaceGen to change the facial features of a single prototypical face. “Although artificial faces may allow greater experimental control over trustworthiness level, they are often perceived as unnatural and are often confused with one another because they are usually derived from a limited number of prototypical face models” (Lischke et al., 2017). Thus, computer-generated faces might affect the results to some extent. It is not clear whether the results will change when more ecological stimuli are used. Another problem with the face set was that trustworthy faces were similar to happy faces and untrustworthy faces were similar to angry faces, but facial dominance was not affected by facial expressions (Oosterhof and Todorov, 2008; Engell et al., 2010). However, previous studies on trustworthiness did not control the valence and arousal of faces. Electroencephalogram (EEG) studies found that the late positive potential (LPP) differences between trustworthy and untrustworthy faces were similar to the LPP differences between happy and angry faces (Schupp et al., 2004; Lischke et al., 2017). Thus, the effects of facial trustworthiness on suppression times might actually reflect the preconscious processing of facial expressions. It is necessary to control valence and arousal when exploring the processing of facial dominance and trustworthiness. Additionally, it is not clear whether the effect of dominance and trustworthiness on preconscious processing is the same as that on computer-generated faces when using real faces as stimuli (Lischke et al., 2017). However, no previous study has used real faces to explore the preconscious processing of facial dominance and trustworthiness.

Furthermore, previous studies showed that for male and female faces, the perception of trustworthiness is different. Dzhelyova et al. (2012) used event-related potentials (ERPs) to investigate whether the gender of a face modulated the temporal dynamics of trustworthiness attribution. They asked participants to judge whether the target face was trustworthy or not. Their results showed that untrustworthy male faces increased the negativity of N170 amplitude and the amplitude of early posterior negativity (EPN; 230−280 ms) compared with trustworthy male faces. In contrast, trustworthy female faces elicited a larger negativity of N170 amplitude and EPN amplitude than did untrustworthy female faces. In economic decision-making tasks, participants considered female faces to be more trustworthy, and they were more willing to choose females as partners and shared more money with female partners (Carragher et al., 2017). In addition, the influence of dominance on behavior is regulated by the gender of a face. People’s stereotypes reflected that dominance was more in line with the characteristics of men than the characteristics of women (Oh et al., 2019). The more dominant a male face was, the more competent he was. However, the more dominant a female face was, the less feminine she was (Wang et al., 2018). Dominant female faces were judged to be more negative than dominant male faces (Sutherland et al., 2015). Furthermore, Mattarozzi et al. (2015) asked participants to evaluate the trustworthiness of faces and found that female participants’ ratings were higher for trustworthy faces than were male participants’ ratings. However, it is not clear whether this moderating effect of gender can occur during the preconscious processing of facial dominance and trustworthiness. Therefore, considering that both face gender and participant gender can influence the preconscious processing of facial trustworthiness and dominance as well as its effect on behavior, we added these two independent variables in the present study.

In summary, although much progress has been made in research on the preconscious processing of facial trustworthiness and dominance, there is still a lack of understanding with regard to this preconscious processing. Previous studies have used computer-generated face stimuli, which did not reveal the role of face gender in the preconscious processing of trustworthiness and dominance. Therefore, some issues still need to be further studied. For example, are there differences in the preconscious processing of trustworthiness and dominance between male and female faces? Can the gender of participants influence the preconscious processing of trustworthiness and dominance? Adopting real Chinese face images as stimuli, the present study used the b-CFS paradigm to investigate whether there were differences in the preconscious processing of faces with different genders and different levels of trustworthiness and dominance in Chinese participants. In the face rating experiment, first, for the face stimuli, we selected three levels across two dimensions, that is, the least, neutral and the most trustworthy/dominant faces; there was no significant difference in arousal and pleasure. Second, Experiments 1 and 2 explored the preconscious processing of facial trustworthiness and dominance, respectively, as well as the influence of face gender and participant gender on such processing.

## Face Rating Experiment

### Method

#### Participants

Thirty-four participants (17 females, *M*_*age*_ = 21.56, *SD*_*age*_ = 2.12) were recruited from Liaoning Normal University. All participants were physically and mentally healthy, with normal or corrected-to-normal vision, and without color blindness or color weakness. None of them had participated in any similar experiments before, and they had never seen the face stimuli. All participants were paid at the end of the experiment. One participant was excluded from the analysis for not understanding the instructions of the experiment. The final sample consisted of 33 participants (16 males and 17 females).

#### Apparatus and Stimuli

The experimental paradigm was programed using E-Prime 2.0 and was run on a Lenovo desktop. The stimuli were presented on a 19-inch LCD monitor (1440 × 900 pixels) at a viewing distance of 57 cm.

**FIGURE 1:**
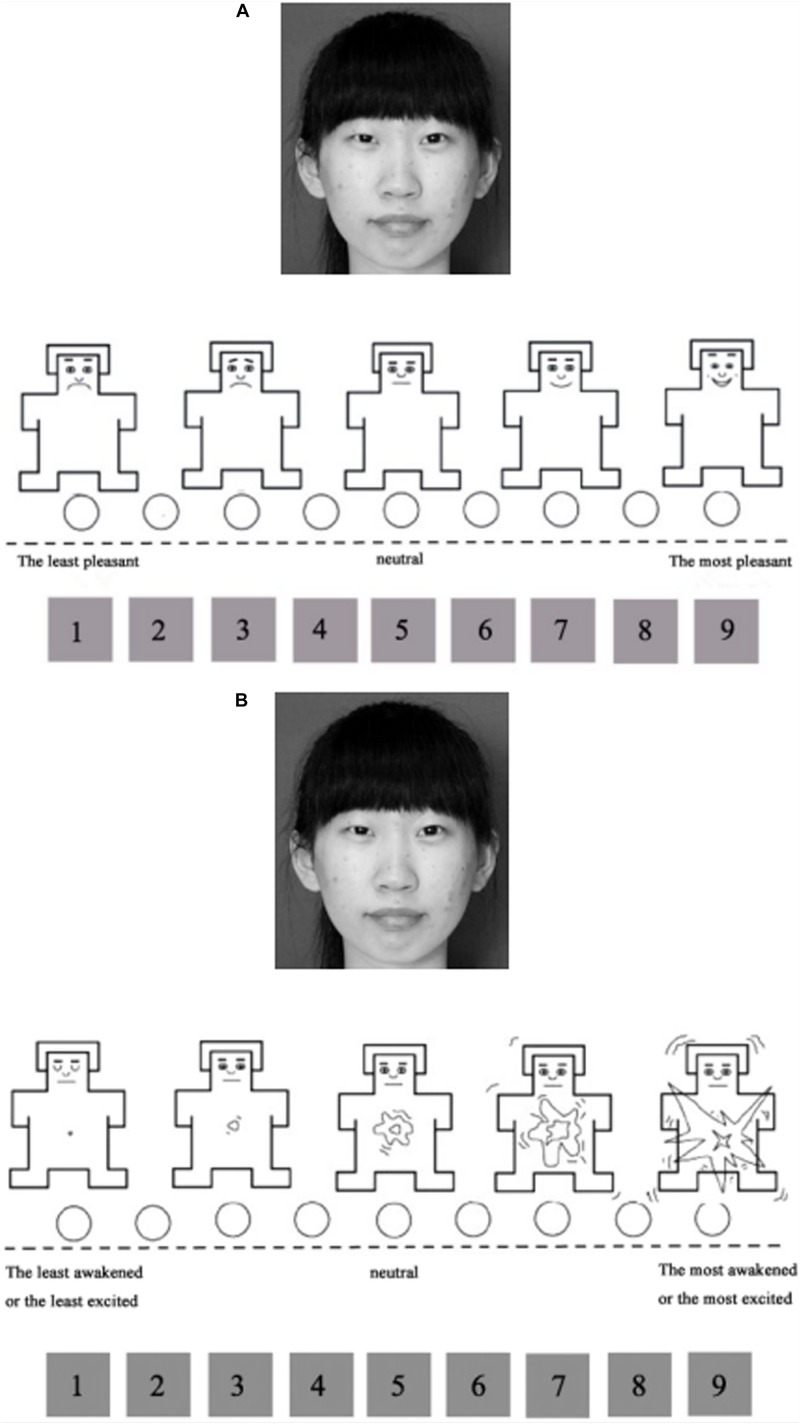
**(A)** The rating scale for pleasure. A face and the scale were presented at the same time. The characteristics of the cartoon figures in the picture represented different levels of pleasure. Regarding the numbers below the face, 1 indicated that the face made the participant feel the most unpleasant, 5 meant neutral, and 9 meant the face made the participant feel the most pleasant. **(B)** The rating scale for arousal. A face and the scale were presented at the same time. The characteristics of the cartoon figures in the picture represented different levels of arousal. Regarding the numbers below the face, 1 indicated that the face made the participant feel the least awakened, excited, and tense, 5 meant neutral, and 9 meant that the face made the participant feel the most awakened, excited, and tense.

The stimuli were 244 photos that were collected on the Internet (125 males and 119 females). The images had previously been cropped and processed into grayscale using Photoshop 8.0.1. We programed in Visual Basic and calculated the average RMS contrast of all the images, then adjusted the contrast of each image to the average value. The visual angle of all images was approximately 4.2° × 6.7°, and the screen background was gray (RGB: 128, 128, 128). A 9-point Self-Assessment Manikin (SAM; Bradley and Lang, 1994) scale was used to rate the pleasure and arousal of the faces, as shown in Figure 1.

#### Procedure

The experiment included three tasks. First, the participants needed to evaluate facial trustworthiness. Then, they were asked to evaluate facial dominance. Third, they were asked to evaluate the pleasure and arousal of the faces. In task 1 and task 2, the participants were asked to click the corresponding number with the left mouse button to rate the dominance and trustworthiness of faces on a 7-point Likert scale (1: the least trustworthy/dominant, 4: neutral, 7: the most trustworthy/dominant). In task 3, a face and a SAM scale appeared on the screen at the same time. The participants needed to evaluate pleasure and arousal based on their gut feeling about the face (see Figure 1). Practice trials preceded each task, and the order of tasks was counterbalanced across participants.

### Results

#### The Selection of Faces With Different Levels of Trustworthiness

The average ratings of dominance, trustworthiness, arousal and pleasure of each face were calculated based on the data of the 33 participants. We also caculated the inter-rater reliability for the trustworthiness, dominance, arousal, and pleasure ratings. The Kendall’s W for trustworthiness, dominance, arousal, and pleasure ratings is 0.252, 0.188, 0.200, 0.362. First, we sorted the trustworthiness ratings from low to high; then, we selected faces with no significant difference in arousal and pleasure; and, finally, we selected the most representative faces. A total of 66 faces were ultimately selected. They were divided into three groups according to the trustworthiness ratings, that is, the least trustworthy group, the neutral group, and the most trustworthy group. There were 22 images in each group, with half the faces being male faces and the other half being female faces. The descriptive values are shown in Table 1.

**TABLE 1:** Mean and standard deviation of trustworthiness, dominance, arousal, pleasure, and contrast of the 66 faces.

**Trustworthiness levels**	**Trustworthiness**		**Dominance**		**Arousal**		**Pleasure**		**Contrast**	
	***M***	***SD***	***M***	***SD***	***M***	***SD***	***M***	***SD***	***M***	***SD***
The least trustworthy	3.14	0.30	3.63	0.58	4.14	0.43	3.84	0.50	52.81	4.68
Neutral	3.60	0.36	3.56	0.63	4.39	0.59	3.91	0.57	52.06	11.92
The most trustworthy	4.05	0.33	3.60	0.52	4.32	0.47	3.77	0.52	52.33	4.44
										

Five separate one-way ANOVAs were performed on the trustworthiness, dominance, arousal, pleasure and contrast ratings. There were significant differences in trustworthiness among the three groups, *F*(2, 63) = 41.08, *p* < 0.001, η_p_^2^ = 0.57. *Post hoc* analysis (Bonferroni corrected) showed that there were significant differences between the least trustworthy faces and neutral faces (*p* < 0.001, 95% CI [−0.70, −0.21]), between the most trustworthy faces and neutral faces (*p* < 0.001, 95% CI [−0.70, −0.21]), and between the least and the most trustworthy faces (*p* < 0.001, 95% CI [−1.15, −0.66]). There was no significant difference in dominance [*F*(2, 63) = 0.09, *p* = 0.916], arousal [*F*(2, 63) = 1.43, *p* = 0.247], pleasure [*F*(2, 63) = 0.36, *p* = 0.697] or contrast [*F*(2, 63) = 0.05. *p* = 0.95].

#### The Selection of Faces With Different Levels of Dominance

The same procedure was used to divide the faces into three groups: the least dominant group, the neutral group and the most dominant group. A total of 66 faces were ultimately selected. There were 22 images in each group, with half the faces being male faces and the other half being female faces (see Table 2 for descriptive values). Additionally, there were 26 faces used in both experiments.

**TABLE 2:** Mean and standard deviation of dominance, trustworthiness, arousal, pleasure, and contrast of the 66 faces.

**Dominance levels**	**Dominance**		**Trustworthiness**		**Arousal**		**Pleasure**		**Contrast**	
	***M***	***SD***	***M***	***SD***	***M***	***SD***	***M***	***SD***	***M***	***SD***
The least dominant	2.81	0.19	3.40	0.53	3.68	0.29	3.93	0.67	52.51	4.49
Neutral	3.55	0.03	3.48	0.49	3.78	0.42	4.13	0.72	53.19	5.76
The most dominant	4.21	0.31	3.55	0.48	3.91	0.38	4.28	0.59	52.96	4.43

Five separate one-way ANOVAs were performed on the dominance, trustworthiness, arousal, pleasure and contrast ratings. There were significant differences in dominance, *F*(2, 63) = 242.10, *p* < 0.001, η_p_^2^ = 0.89. *Post hoc* analysis (Bonferroni corrected) showed that there were significant differences between the least dominant faces and neutral faces (*p* < 0.001, 95% CI [−0.90, −0.58]), between the most dominant faces and neutral faces (*p* < 0.001, 95% CI [−0.82, −0.50]), and between the least and the most dominant faces (*p* < 0.001, 95% CI [−1.55, −1.24]). No significant difference was found in trustworthiness [*F*(2, 63) = 0.49, *p* = 0.617], arousal [*F*(2, 63) = 2.12, *p* = 0.128], pleasure [*F*(2, 63) = 1.58, *p* = 0.214] or contrast [*F*(2, 63) = 0.11, *p* = 0.897].

## Experiment 1: The Role of Face Gender and Participant Gender in the Preconscious Processing of Trustworthiness

### Method

#### Participants

Forty-six college students (27 females, *M*_*age*_ = 22.8, *SD*_*age*_ = 3.34 years) were recruited from Liaoning Normal University and were paid. In addition to the same participant requirements as those in the pilot experiment, all participants were right-handed and without amblyopia and strabismus. One participant was excluded from the analysis because the program crashed during the experiment. For further analysis, the final sample consisted of 45 participants (19 males and 26 females).

#### Design

The experiment used a 3 (trustworthiness: the least trustworthy, neutral, the most trustworthy) × 2 (face gender: male, female) × 2 (participant gender: male, female) mixed design. Trustworthiness and face gender were within-subject variables. Participant gender was the between-subjects variable. Suppression time was the dependent variable.

#### Apparatus and Stimuli

The experimental paradigm was programed using E-Prime 2.0 and was run on an HP 280 Pro G2 MT desktop. The stimuli were presented on a 19-inch LCD monitor (1440 × 900 pixels) with a refresh rate of 60 Hz. A mirror stereoscope was used to reflect the stimuli on both sides of the screen to the left and right eyes of the participants.

The background of the screen was gray (RGB: 128, 128, 128). On the left and right sides of the screen, there were two square gray frames (10.65° × 10.65°, RGB: 128, 128, 128) with black edges symmetrical to the center of the screen. Two black fixations (1.26° × 1.26°) were located at the center of the frame. All stimuli appearing in the experiment were presented inside of the frame. The 66 faces with three levels of trustworthiness that were selected from the pilot experiment were used as original pictures. Photoshop 8.0.1 was used to create pictures with 10, 20, 30, 40, 50, 60, 70, 80, and 90% of the transparency of the original pictures (Stein et al., 2017). The created pictures were used as target stimuli along with the original pictures (i.e., transparency was 0); when the transparency was 100%, the picture was the background color of the frame. Ten chromatic Mondrian noises were generated by MATLAB 7.0.

#### Procedure

Before the experiment, the Dolman method (Anderson et al., 2012) was used to determine the dominant eye of the participants. The experiment was carried out in a dark and quiet room. The participants were comfortably seated in a chair, with their eyes approximately 57 cm from the screen; their chins were fixed on a chin rest. By properly calibrating and adjusting the mirror stereoscope, the images of the left and right visual fields overlapped well in the center.

**FIGURE 2:**
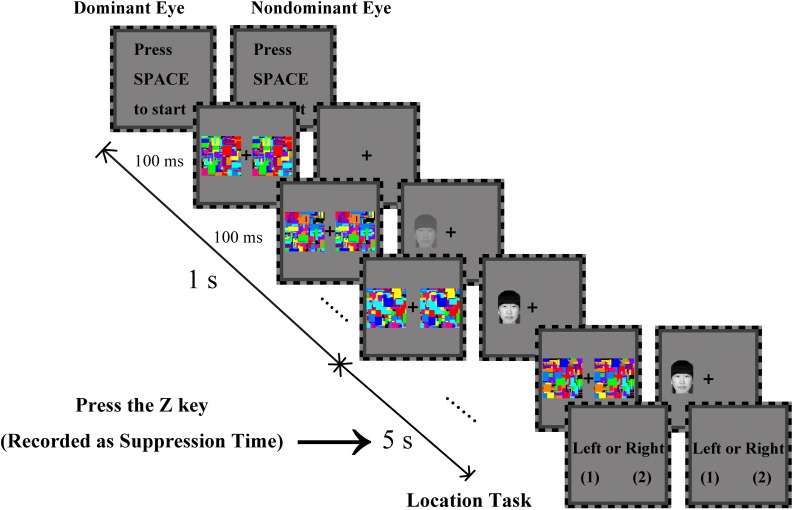
Procedure of Experiment 1 and Experiment 2. Chromatic noises (frequency 10 Hz) were presented to the dominant eye. Faces were presented to the non-dominant eye, and they appeared randomly on the left or right side of the fixation. The transparency of the face linearly decreased from 100 to 0% over the span of 1 s and subsequently remained constant for the next 5 s until the participant responded. The participants were instructed to press the *Z* key as soon as any part of the face was detected. The stimulus disappeared, and the location task was then presented until the participant pressed the *Z* key or did not respond within 6 s. To facilitate understanding, the minimum transparency of the faces was 30%.

The experimental paradigm is shown in Figure 2. Each trial began with the instruction “Press the space bar to continue”. Two identical dynamic chromatic noises (3.68° × 3.68°) appeared on both sides of the fixation in the dominant eye and changed every 100 ms (frequency 10 Hz). At the same time, a face (1.84° × 2.83°) with gradually decreasing transparency (Jiang et al., 2007; Stein et al., 2017) appeared either on the left or the right of fixation in the non-dominant eye (the distance between the face center and the fixation was 2.32°). In the trial, the transparency of the face decreased from 100 to 0% linearly by a 10% decrement every 100 ms over the span of 1 s and subsequently remained constant for the next 5 s until the participant responded. The participants were asked to press the *Z* key on a standard keyboard as soon as any part of the face was detected; they were asked to respond as accurately and quickly as possible. The time from the start of each trial (after pressing the space bar) to the time when the *Z* key was pressed was recorded as the suppression time. The participants were then instructed to press the 1 or 2 key to indicate whether the face was on the left or right side of the fixation. If the participant did not respond within 6 s, the trial would end. There was a practice block of 30 trials before the formal experiment. The experiment consisted of 528 trials (8 blocks of 66 trials each). Each face appeared only once in each block; in each block, the 66 faces were equally likely to appear on the left or the right side of the fixation, there were 176 trials performed by each participant for each trustworthiness category. The order of trials of each block was randomized.

### Results

The data of the 45 participants were analyzed. The suppression times for wrong responses in the face location task were excluded. Suppression times less than 100 ms and more than 6000 ms were also excluded because the face did not appear within 100 ms and disappeared after 6000 ms (all excluded data accounted for 2.47% of the total data). The average suppression time of each participant for the least trustworthy, neutral and the most trustworthy faces of different genders was calculated.

**FIGURE 3:**
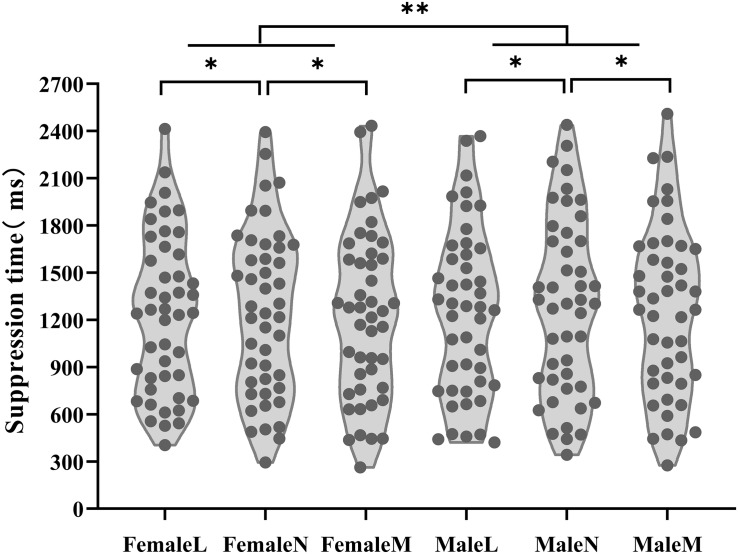
The results of Experiment 1: The plot shows the suppression time of the participants for the least trustworthy, neutral and the most trustworthy faces of different genders (male and female faces). ^∗^*p* < 0.05, ^∗∗^*p* < 0.01. In the figure, FemaleL means the female least trustworthy faces, FemaleN means the female neutral faces, FemaleM means the female most trustworthy faces, MaleL means the male least trustworthy faces, MaleN means the male neutral faces, MaleM means the male most trustworthy faces.

A 2 (face gender: male, female) × 3 (trustworthiness: the least trustworthy, neutral and the most trustworthy) × 2 (participant gender: male, female) ANOVA was applied to the suppression time. The assumption of sphericity was met. In addition, for mean suppression times from the b-CFS task we conducted Bayesian analyses using JASP (JASP Team, 2019). Bayes factors (BFs) were calculated to quantify the evidence for the presence or the absence of a main effect of face gender, trustworthiness and participant gender in a Bayesian repeated-measures ANOVA, which was followed up with Bayesian paired *t*-tests to compare the three levels in trustworthiness, using the JASP default settings (Cauchy prior width 0.707) (Stein et al., 2017). Furthermore, we followed Wetzels and Wagenmakers (2012) to assign categorical labels to BFs. We labeled BFs between 1 and 3 “anecdotal evidence,” BFs between 3 and 10 “substantial evidence,” BFs between 10 and 30 “strong evidence,” and BFs between 30 and 100 “very strong evidence.” Moreover, the sequential Bonferroni correction was applied to the significance level of the ANOVA and *t*-tests (see Tables 3, 4). As shown in Figure 3, the analysis revealed a marginally significant main effect of trustworthiness, *F*(2,86) = 4.82, *p* = 0.01, η_p_^2^ = 0.10, *BF*_10_ = 27.008. Paired-sample *t*-tests showed that the participants were significantly slower to respond to neutral faces (*M* = 1266 ms, *SD* = 543 ms) than to the least trustworthy faces (*M* = 1243 ms, *SD* = 514 ms) (*t*(44) = 2.63, *p* = 0.012, Cohen’s *d* = 0.39, 95% CI [6 ms, 42 ms], *BF*_10_ = 3.181) and the most trustworthy faces (*M* = 1240 ms, *SD* = 534 ms) (*t*(44) = 2.48, *p* = 0.017, Cohen’s *d* = 0.37, 95% CI [5 ms, 47 ms], *BF*_10_ = 2.413). There was no significant difference in suppression time between the least trustworthy faces and the most trustworthy faces, *t*(44) = 0.25, *p* = 0.802, 95% CI [−17 ms, 22 ms], *BF*_10_ = 0.167. The main effect of face gender was significant, *F*(1,43) = 7.97, *p* = 0.007, η_p_^2^ = 0.16, *BF*_10_ = 1.232. The suppression times for male faces (*M* = 1264 ms, *SD* = 542 ms) were significantly longer than those for female faces (*M* = 1236 ms, *SD* = 518 ms). The main effect of participant gender was not significant, *F*(1,43) = 4.42, *p* = 0.041, η_p_^2^ = 0.09, *BF*_10_ = 0.876. The other effects and interactions were not significant, *F*s < 2.73, *p*s > 0.07, *BF*_10_ < 1.

**TABLE 3:** The results from the sequential Bonferroni correction of seven different *F* tests of Experiment 1.

**Effect**	***p*-value**	**α_adj_**	**H_0_**

Face gender	0.007	0.0071	rejected
Trustworthiness	0.010	0.008	rejected (marginally significant)
Participant gender	0.041	0.01	retained
			

**TABLE 4:** The results from the sequential Bonferroni correction of three different *t*-tests of Experiment 1.

**Effect**	***p*-value**	**α_adj_**	**H_0_**

The least trustworthy faces and neutral faces	0.012	0.016667	rejected
The most trustworthy faces and neutral faces	0.017	0.025	rejected

An independent sample *t*-test was performed on the accuracy of male and female participants in Experiment 1. The results showed the difference in accuracy between male and female participants was not significant, *t*(43) = −0.389, *p* = 0.70.

According to Stein et al. (2018), the differences of the high and low trustworthiness to the neutral category were calculated and the results were further analyzed. A 2 (difference type: the least trustworthy-neutral, the most trustworthy-neutral) × 2 (face gender: male, female) × 2 (participant gender: male, female) ANOVA was applied to the suppression time difference. The interaction of difference type and participant gender was significant, *F*(1,34) = 5.68, *p* = 0.022, η_p_^2^ = 0.12. The other effects and interactions were not significant, *F*s < 1, *p*s > 0.05. Then, simple effect analysis was performed for the interaction. The effect of the participant gender on the two difference types was not significant (the least trustworthy-neutral: *F*(1,34) = 0.60, *p* = 0.443, the most trustworthy-neutral: *F*(1,34) = 2.03, *p* = 0.162). At the same time, for male and female participants, paired sample *t*-test was performed on the suppression time difference for difference types, for male and female participants, difference type had no significant effect on the suppression time difference (male participants: *t*(18) = 1.91, *p* = 0.072, female participants: *t*(25) = −1.39, *p* = 0.178). In summary, the results showed that the differences of the most or least trustworthiness and the neutral trustworthiness were similar.

### Discussion

Experiment 1 showed that the suppression time for neutral faces was significantly longer than that for the least and the most trustworthy faces, which was different from the results of Stewart et al. (2012) and Stein et al. (2018). In their studies, the response time for the least trustworthy faces was significantly longer than that for neutral faces. However, Abir et al. (2018) revealed that the more untrustworthy a face was, the shorter the suppression time, which is partially similar to the current experiment. Trustworthiness symbolizes whether someone intends to harm others (Todorov et al., 2008b). However, Experiment 1 showed that the least trustworthy faces did not cause a passive fear response, which was proposed by Stewart et al. (2012), but accelerated preconscious processing. Therefore, the participants responded more quickly to the least trustworthy faces at the preconscious level. In addition, the most trustworthy faces broke through suppression significantly more quickly than the neutral faces. The reason may be that trustworthy faces contribute to cooperation; people tend to choose a trustworthy person as an investment partner (Wout and Sanfey, 2008; Stirrat and Perrett, 2010; Rezlescu et al., 2012). Thus, trustworthy faces are processed preconsciously faster than neutral faces.

In addition, Experiment 1 also found that female faces broke through suppression significantly more quickly than male faces. The reason may be that cooperation and collective triumph are advocated in China. Compared with men, women are more cooperative (Carragher et al., 2017); thus, female faces took significantly less time to break through suppression. Although trustworthiness is the main premise of cooperation, the strength of the partner should also be considered. Dominance symbolizes competence (Todorov et al., 2008b; Wang et al., 2018). When people choose team members, they tend to choose people with the most dominant faces (Hehman et al., 2015). Therefore, Experiment 2 was performed to explore the influence of face gender and dominance on preconscious processing in Chinese culture.

## Experiment 2: The Role of Face Gender and Participant Gender in the Preconscious Processing of Dominance

### Method

#### Participants

Fifty-three college students (27 females, *M*_*age*_ = 21.34, *SD*_*age*_ = 2.04 years) were recruited from Liaoning Normal University and were paid. The participant requirements were the same as those in Experiment 1. Three participants were excluded from the analysis (one participant’s accuracy was less than 60%, and the program crashed when the other two participants were completing the experiment). The final sample consisted of 50 participants (25 males and 25 females).

#### Design

The design was the same as that in Experiment 1, except that the independent variable was dominance (the least dominant, neutral, the most dominant) instead of trustworthiness.

#### Apparatus and Stimuli

Faces with different levels of dominance that were selected from the pilot experiment were used as stimuli in Experiment 2. All other aspects were the same as those in Experiment 1. Additionally, 26 faces were used in both Experiment 1 and Experiment 2.

#### Procedure

All procedures were the same as those in Experiment 1, as shown in Figure 2.

### Results

The data were analyzed and excluded in the same way as in Experiment 1 (all excluded data accounted for 2.82% of the total data). The average suppression times of each participant for the least dominant, neutral and the most dominant faces of different genders were calculated.

**FIGURE 4:**
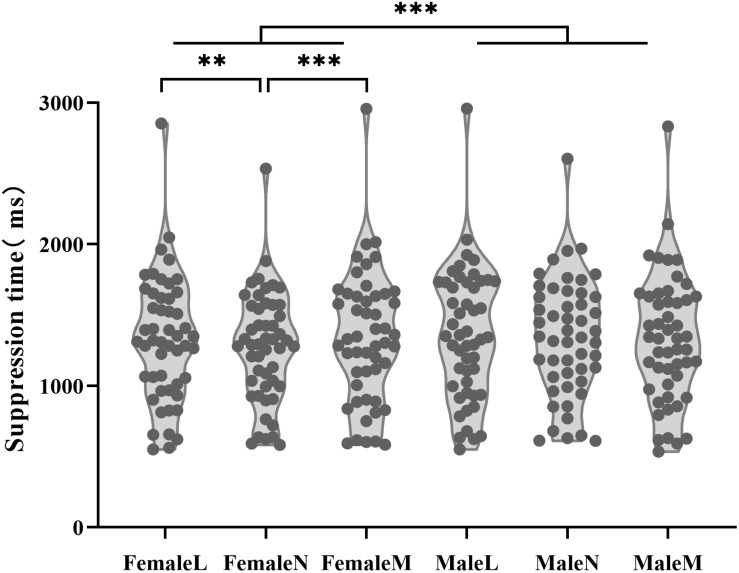
The results of Experiment 2: The plot shows the suppression time of the participants for the least dominant, neutral and the most dominant faces of different genders (male and female faces). ^∗∗^*p* < 0.01, ^∗∗∗^*p* < 0.001. In the figure, FemaleL means the female least dominant faces, FemaleN means the female neutral faces, FemaleM means the female most dominant faces, MaleL means the male least dominant faces, MaleN means the male neutral faces, MaleM means the male most dominant faces.

A 2 (face gender: male, female) × 3 (dominance: the least dominant, neutral and the most dominant) × 2 (participant gender: male, female) ANOVA was applied to the suppression time. Since the assumption of sphericity was violated, the Greenhouse-Geisser correction was used. We also applied Bayes Factor method as same as Experiment 1. The sequential Bonferroni correction was applied to the significance level of the ANOVA and *t*-tests results (see Tables 5–7). The analysis revealed a marginally significant main effect of dominance, *F*(1.66, 79.84) = 5.28, *p* = 0.011, η_p_^2^ = 0.10, *BF*_10_ = 40.390. A significant main effect of face gender was also found, *F*(1,48) = 15.09, *p* < 0.001, η_p_^2^ = 0.24, *BF*_10_ = 8.115; female faces (*M* = 1310 ms, *SD* = 430 ms) took significantly less time to break through suppression than male faces (*M* = 1341 ms, *SD* = 440 ms). The interaction between dominance and face gender was significant, *F*(1.68,80.67) = 6.50, *p* = 0.004, η_p_^2^ = 0.12, *BF*_10_ = 8.558. The simple effect analysis showed that for males faces, the main effect of dominance was not significant, *F*(2,98) = 2.15, *p* = 0.122, η_p_^2^ = 0.04, *BF*_10_ = 0.399. For female faces, the main effect of dominance was significant, *F*(2,98) = 10.19, *p* < 0.001, η_p_^2^ = 0.17, *BF*_10_ = 225.722. Paired-sample *t*-tests showed that the least dominant female faces (*M* = 1325 ms, *SD* = 442 ms) broke through suppression significantly more slowly than neutral female faces (*M* = 1272 ms, *SD* = 389 ms), *t*(49) = 3.46, *p* = 0.001, Cohen’s *d* = 0.49, 95% CI [22 ms, 84 ms], *BF*_10_ = 25.361. The most dominant female faces (*M* = 1333 ms, *SD* = 463 ms) also took significantly longer to break through suppression than neutral female faces, *t*(49) = 4.03, *p* < 0.001, Cohen’s *d* = 0.57, 95% CI [30 ms, 91 ms],*BF*_10_ = 123.963. There was no significant difference in suppression times between the least dominant and the most dominant female faces, *t*(49) = 0.56, *p* = 0.577, 95% CI [−19 ms, 34 ms], *BF*_10_ = 0.179. The other effects and interactions were not significant, *F*s < 2.41, *p*s > 0.10, *BF*_10_ < 1. The results are shown in Figure 4.

**TABLE 5:** The results from the sequential Bonferroni correction of seven different *F* tests of Experiment 2.

**Effect**	***p*-value**	**α_adj_**	**H_0_**

Face gender	<0.001	0.0071	rejected
Dominance × face gender	0.004	0.008	rejected
Dominance	0.011	0.01	rejected (marginally significant)
			

**TABLE 6:** The results from the sequential Bonferroni correction of *F* tests for female faces of Experiment 2.

**Effect**	***p*-value**	**α_adj_**	**H_0_**

Dominance	<0.001	0.05	rejected
			

**TABLE 7:** The results from the sequential Bonferroni correction of three different *t*-tests for female faces of Experiment 2.

**Effect**	***P*-value**	**α_adj_**	**H_0_**

The most dominant female faces and neutral female faces	<0.001	0.01667	rejected
The least dominant female faces and neutral female faces	0.001	0.025	rejected

Similar with Experiment 1, the difference of the high and low dominance to the neutral category was calculated. A 2 (difference type: the least dominant-neutral, the most dominant-neutral) × 2 (face gender: male, female) × 2 (participant gender: male, female) ANOVA was applied to the suppression time difference, the Greenhouse-Geisser correction was used. The main effect of face gender was significant, *F*(1,48) = 15.06, *p* < 0.001, η_p_^2^ = 0.24, the interaction of face gender and participant gender was significant, *F*(1,48) = 7.69, *p* = 0.022, η_p_^2^ = 0.01. Then for male participants, paired sample *t*-test was performed on the difference suppression time for difference face gender, the results showed the suppression time difference of female faces was significantly greater than that of the male faces, *t*(24) = −4.84, *p* < 0.001. For female participants, there was no significant difference in difference suppression time between male faces and female faces, *t* (24) = −0.763, *p* = 0.453. For both male faces and female faces, paired sample *t*-test was performed on the difference suppression time for different participants gender, the results showed the suppression time differences were similar between male participants and female participants (male faces:*t* (24) = −1.46, *p* = 0.157, female faces:*t* (24) = −1.34, *p* = 0.193). The other effects and interactions were not significant, *F*s < 3, *p*s > 0.05. In summary, the results did not show effect of dominance.

### Discussion

Experiment 2 showed that dominance had different effects on the suppression time for faces of different genders. There was no significant difference in suppression times for male faces in different dominance level. For female faces, neutral faces broke through suppression more quickly than the least and the most dominant faces. Some research (Stewart et al., 2012; Getov et al., 2015; Stein et al., 2018) has found that the most dominant faces took significantly longer to break through suppression than neutral faces; however, Abir et al. (2018) have suggested that the more dominant a face was, the shorter the suppression time it took. Stewart et al. (2012) suggested that similar to angry faces, the most dominant faces posed a threat, causing passive fear responses to slow visual perception for participants. The previous studies were conducted in Great Britain (Stewart et al., 2012; Getov et al., 2015), and Israel (Abir et al., 2018). The current study was conducted in China. Cultural differences could have an influence on the awareness of facial dominance. Because cooperation is emphasized in Chinese culture, faces that benefit cooperation may facilitate preconscious processing. The least dominant female face reflects a low level of competence and means little with regard to cooperation; thus, the preconscious processing of the least dominant female faces was slower. In contrast, females with neutral faces are more likely to be chosen as partners; thus, they were processed preconsciously faster. However, people tend to think that the more dominant a female face is, the more masculine this female is (Wang et al., 2018; Oh et al., 2019), and they tend to have a negative impression of dominant females (Sutherland et al., 2015). Therefore, the most dominant female faces took significantly longer to break through suppression than neutral female faces.

## General Discussion

Using real faces, the present research explored the effect of face gender and participant gender on the preconscious processing of trustworthiness and dominance. The results were different from previous studies using computer-generated faces (Stewart et al., 2012; Getov et al., 2015; Stein et al., 2018). Experiment 1 found that both the least and the most trustworthy faces took significantly less time to break through suppression than neutral faces. In contrast, Stewart et al. (2012) and Stein et al. (2018) found that the least trustworthy faces broke through suppression significantly more slowly than neutral faces. The following reasons may explain this inconsistency. First, Stewart et al. (2012) suggested that the preconscious processing of trustworthiness was influenced by interpersonal trust, which might be different across participants. However, Stein et al. (2018) failed to replicate the effect of interpersonal trust. Since the current study did not measure the interpersonal trust propensity of participants, it remains unclear whether individual differences cause the discrepancy between our results and those of Stewart et al. (2012) and Stein et al. (2018). Second, trustworthiness symbolizes whether a stranger intends to harm others; people tend to approach trustworthy faces and avoid untrustworthy faces (Todorov et al., 2008a). Thus, people are more sensitive to the least trustworthy faces, which is consistent with Abir et al. (2018). Furthermore, Marzi et al. (2012) asked participants to make a decision about whether they would vote for the presented face and to evaluate how much they trusted the face. Both tasks showed that it took participants less time to evaluate the least trustworthy faces compared with the most trustworthy and neutral faces; additionally, the accuracy was higher. Consistent with these previous results, our study also found that the least trustworthy faces broke through suppression significantly more quickly than neutral faces. Third, participants tend to believe people with more trustworthy faces and invest more money with them in investment decision-making (Wout and Sanfey, 2008; Stirrat and Perrett, 2010; Rezlescu et al., 2012). It can be seen that the most trustworthy faces are conducive to cooperation. Chinese people attach great importance to cooperation, and facial traits that contribute to cooperation receive prioritized processing. Therefore, the most trustworthy faces took significantly less time to break through suppression than neutral faces.

In Experiment 2, the main effect of dominance was only found for female faces, neutral faces broke through suppression significantly more quickly than the least and the most dominant faces. However, Stewart et al. (2012), Getov et al. (2015), and Stein et al. (2018) found that there were no significant differences between the least dominant faces and neutral faces. Moreover, different from the current study, they found that the most dominant faces took significantly longer to break through suppression than neutral faces. There are possible reasons for the inconsistency between the results of our study and theirs. First, the difference of the stimulus material might be an important aspect of the inconsistency. Very different from previous studies (Stewart et al., 2012; Getov et al., 2015; Stein et al., 2018), we got results based on actual faces, there were differences in facial characteristics between real faces and computer-generated faces (Dyck et al., 2008; Mühlberger et al., 2009). Unlike computer-generated faces, physical characteristics of the real face cannot be controlled. Besides, real faces that meet the requirement are hard to select and can’t be flexibly manipulated to an exact combination of levels on both dominance and trustworthiness dimension. However, using real faces added to the practical significance of the study. Second, cooperation and harmonious social relations are encouraged in collectivistic cultures (Freeman et al., 2009). China is collectivistic culture. In general, neutral female faces are the most cooperative faces; thus, they broke through suppression faster than other faces. A previous study found that the more dominant a female face was, the more negative the impression it gave to participants; even when the face was presented only 500 ms, this effect persisted (Sutherland et al., 2015). People might also find that dominant women are difficult to work with; thus, the most dominant female faces broke through suppression more slowly than neutral female faces. Furthermore, Chiao et al. (2008) also found that compared with neutral faces, the amplitude of N200 induced by the least dominant faces was significantly smaller. The larger the amplitude of N200, the more attentional resources were allocated to the faces (Chen et al., 2012). For female faces, since less attention was paid to the least dominant faces, they were perceived preconsciously more slowly.

In addition, both experiments found that female faces broke through suppression significantly more quickly than male faces. In investment decision-making, people are more willing to trust women than men (Carragher et al., 2017). Cooperation is encouraged in China. When people choose cooperation partners, the trustworthiness and dominance cues in female faces may be more obvious than those in male faces, which might explain why it took less time for female faces to break through suppression.

Furthermore, the data for the relevant previous studies were assessed in Great Britain (Stewart et al., 2012; Getov et al., 2015), Israel (Abir et al., 2018), and Italy (Stein et al., 2018). The present research was conducted in China. Cultural differences could have an influence on the awareness of facial trustworthiness and dominance. However, since we did not compare participants from different cultural backgrounds, it is unclear whether culture plays a role. Future studies could make directly cross-cultural comparisons on the processing of facial trustworthiness and dominance.

There are several limitations in the current study. Firstly, although the results showed significant differences in response times in b-CFS, they were insufficient to claim high-level unconscious processing. There is an ongoing debate whether response times measured during b-CFS provide evidence for unconscious processing at all (e.g., Moors et al., 2017, 2019). Moors et al. (2019) argued that very few studies showed high-level unconscious processing in b-CFS if the proper controls were included. Future research should use the dissociation approach where an implicit processing measure was contrasted with an explicit awareness measure to claim genuine preconscious processing (Moors et al., 2019). Secondly, many studies found that low-level stimulus differences between conditions in b-CFS might be the major factor that influence the reaction times of faces, instead of facial expressions, trustworthiness or dominance (Gray et al., 2013; Hedger et al., 2015, 2016; Stein et al., 2018). By measuring relative suppression time for scrambled and inverted faces, Abir et al. (2018) investigated to what extent the suppression time could be explained by low-level differences between faces. It should be noted that they did not experimentally control for low-level differences. They showed that low-level visual features are not sufficient to affect the role of social characteristics in unconscious processing of faces. However, these effects were equally strong even when the holistic processing and the perception of social characteristics were impeded by presenting faces upside down. Furthermore, differences in suppression times of different trustworthiness/dominance level can be explained by physical differences in the eye region (Stein et al., 2018). Therefore, low-level properties might weaken the claims of genuine preconscious processing of trustworthiness and dominance in the present study. In addition, the present study used actual photographs of faces, which is a strength of the study in comparison to previous studies using artificial faces, but which also comes at the expense of less controllability of physical characteristics. Future research can improve experimental materials. Finally, it is reasonable to assume that cultural differences could have an influence on the awareness of facial trustworthiness and dominance, future study can address this question.

Another major limitation of the present study is that the effect size of the results is too small. In Experiment 1, the difference in suppression times of different face categories is between 23 and 28 ms. In Experiment 2, the difference in reactions times is between 31 and 47 ms. The reason might be that the difference in ratings of faces between different conditions is small, since the difference in pleasure and arousal was controlled. Future research should apply larger face database to select more distinctive faces which varies more in trust or dominance dimension.

## Data Availability Statement

The datasets generated for this study are available on request to the corresponding author.

## Ethics Statement

The studies involving human participants were reviewed and approved by the IRB of Liaoning Normal University. The participants provided their written informed consent to participate in this study.

## Author Contributions

HW, ST, and JS designed the experiments. HW and ST prepared the materials, performed the experiments, analyzed the data, and wrote the manuscript. JS and WC revised the manuscript.

## Conflict of Interest

The authors declare that the research was conducted in the absence of any commercial or financial relationships that could be construed as a potential conflict of interest.
